# Faces Revealed Project and ancient Egyptian yellow coffins: A new methodology step-by-step

**DOI:** 10.12688/openreseurope.17421.1

**Published:** 2024-09-03

**Authors:** Stefania Mainieri

**Affiliations:** 1 Fondazione Museo Egizio, Torino, Torino, 10123, Italy; 2 Near Eastern Languages & Cultures - UCLA, The University of California, Los Angeles, California, 90095, USA

**Keywords:** Ancient Egypt, yellow coffins, photogrammetry, 3D models, decoration, geometry, facial features

## Abstract

Faces Revealed Project established a new methodology for studying the geometry of the human forms and facial features realized on anthropoid yellow coffins of Ancient Egypt. Since 1980, yellow coffins have been the subject of various studies mainly focused on iconography and palaeography. However, these anthropoid coffins are three-dimensional objects with well-rendered masks and detailed facial features as well as forearms, hands and bellies. This lack of analysis in the study of coffins may be due to the fact that they are “concealed” by rich and multi-coloured decoration, so they are not easily visible in all their forms to the naked eye. Today new technologies allow us to go more in-depth and digitally switch off the decoration and observe these “invisible” features. As this is an entirely new process, the primary task of the Faces Revealed Project was to establish a new methodology from the photogrammetric survey to the data collection.

The present article discusses in detail the stages of the Project applied to around 100 Egyptian yellow coffins stored in Museums in Europe, the United States and Egypt and the information that they can disclose. The task is to share with the scientific community the established protocol and offering the possibility to “work independently” applying the same methodology to the same objects as well as to other classes of material.

## Introduction

Called yellow due to the colour of their background, imitating gold, yellow coffins appeared in Thebes at the end of the New Kingdom (proto-yellow type, from 1279 BCE ca.) and were used for more than a millennium peaking during the 21st Dynasty (1069–945 BCE ca.) ^1^. This type of coffin was crafted in wood and anthropoid in shape, with a detailed rendering of the human forms in the upper part of the lid. They were used in nested funerary assemblages comprising one or two coffins ( 
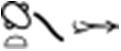
) ^2^ with their lids and cases: a larger outer coffin ( 
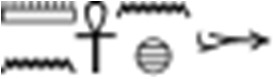
 *mn anx* ) ^3^ containing the inner coffin ( 
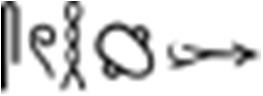
 *swH.t* ) ^4^ that hosted the mummy of the deceased. The “assemblage” of yellow coffins included also a third element, the distinctive part of the yellow coffin type, the mummy board, a flat and small version of the lid placed directly onto the mummy ^5^.

The main characteristic of this type of coffin is the decoration. A coating of varnish over a rich polychrome decoration - characterised by a complete filling of all the empty spaces (horror vacui) - cover both the coffin's external and internal parts. While the pictorial areas on the coffins show a high degree of iconographic autonomy, they are combined into a layout that forms a carefully planned “topography” ^6^. In the Third Intermediate Period (1069–664 BCE ca.) the pictorial and textual tradition of the tomb walls found its way onto the coffins, which came to simultaneously perform the function of the tomb and the temple, thus replacing the burials of earlier periods. We are therefore witnessing what van Walsem calls the “architectonisation” of the coffin. This entails the coffin becoming a small universe, at the centre of which there is the deceased himself, who becomes the source of his own regeneration and rebirth ^7^.

As is easy to understand, images and texts have always been the focus of the numerous studies on yellow coffins, considered the main “diagnostic features” of coffins development between the late New Kingdom and Late Period ^8^. Since the beginning they have been catalogued, typologised and dated by the scholars only on the base of their “Visual appearance” ^9^. The first typology of yellow coffins was made by Andrzej Niwinski in 1988. He collected more than 400 coffins across 88 museums, identifying the yellow type's fundamental characteristics and proposing a typology on the basis of their decoration and layout ^10^. Over the years this fundamental work has been revisited by different scholars such as Rogerio Sousa, Kathlyn Cooney or International Projects, i.e. the Vatican Coffin Project and the Fitzwilliam Museum’s Ancient Egyptian Coffins Project ^11^. The new studies have been focused on correcting some problems in the formal definition of Niwinski’s typology ^12^ and the necessity to create a more precise typology including the ancient reuse of these objects which also led to more in-depth social, economic and religious perspectives ^13^. Furthermore, their materiality has oriented the research to the constituent materials, such as the composition of wood and pigments or the assembly techniques used by artists and craftsmen ^14^.

Still, none of these recent works focused on another fundamental element of the yellow coffin, its anthropoid form which represents the “missing piece” in the Egyptological study of yellow coffins. These coffins, in fact, symbolise the replacement body for the dead ^15^. Shoulders, elbows, forearms, hands, as well as masks with detailed facial features are depicted three-dimensionally. Faces in fact are not only painted on coffins but carefully carved or modelled to allow the deceased to “see, taste, and smell the living world” ^16^. Different from a rectangular coffin, we cannot fail to consider the forms and the geometry of these objects, as well as the way and the style in which the human forms and facial features were crafted. We need to approach this material in the same way as statues and analyse in detail these elements that, if added to the information we already have on these coffins, could help to identify common “hands” or specific stylistic features, clarifying their chronology, especially in the light of the massive reuse of these objects in ancient times ^17^.

Approaching the study of forms and geometry on a polychrome, over-decorated three-dimensional object is hard because the Visual appearance covers its Physical geometry and an objective analysis of forms cannot be made by the naked eye. This limit in geometrical analyses can be overcome today thanks to the digital technologies and 3D modelling. The main instrument of the project is Photogrammetry and its ability to create a real and submillimetric digital reproduction of an object with bi-dimensional images. The resultant 3D model allows us to observe the coffin with and without decoration and analyse the forms accurately without “visual interference”. The ability to create two different visualisations of the same object became the main instrument and the first step of the Faces Revealed Methodology, followed by the possibility to add and overlap in transparency other “layers” mapping both the decoration and the geometry on the model ^18^.

The research is divided into different workpackages and follows a specific methodology: from photogrammetric acquisition to the creation of “layers” and the morphometric approach, data connection and grouping. Starting from the creation of high-resolution and submillimetric photogrammetric models of coffins will be explained in detail all the steps of the methodology and the numerous elements that this analysis can disclose ^19^.

## Methods

### The corpus

The *corpus* of yellow coffins analysed by the Faces Revealed Project consists of more than 100 coffin lids stored in 10 Museums in America, Europe and Egypt ( Table 1) ^20^. The museums' Partners were selected on the basis of pre-existent collaboration and for their significant yellow coffins collections. Moreover, a conspicuous number of these coffins are under investigation by other International Projects for diagnostic investigation and new Egyptological studies, concerned above all with decoration and palaeography as well as the history and provenance of collections ^21^. These in-progress studies and collaborations with different specialists were fundamental in the choice of objects enabling us to combine results with other data acquired from the most current research on yellow coffins ^22^.

**Table 1.** Corpus of the selected yellow coffins.

Museum	Inv. N.	Owner/s	Part of Coffin	Dynasty	Provenance
"Museo Egizio" di Firenze	8521	Ankhsenmut	Mummy Board	21st Dynasty, Middle	Bab el-Gasus Cache
"Museo Egizio" di Firenze	8523	Ankhsenmut	Outer Lid	21st Dynasty, Middle/ Late	Bab el-Gasus Cache
"Museo Egizio" di Firenze	8524	Djedmutiuesankh	Outer Lid	21st Dynasty	Bab el-Gasus Cache
"Museo Egizio" di Firenze	8528	Djedmutiuesankh	Inner Lid	21st Dynasty, Late	Bab el-Gasus Cache
"Museo Egizio" di Firenze	9534	Djedmutiuesankh	Mummy Board	21st Dynasty, Late	Bab el-Gasus Cache
"Museo Egizio" di Firenze	8527	Khonsumes	Inner Lid	21st Dynasty, Middle	Bab el-Gasus Cache
"Museo Egizio" di Firenze	9530	Khonsumes	Mummy Board	21st Dynasty, Middle	Bab el-Gasus Cache
"Museo Egizio" di Firenze	2174	Anonymous	Mummy Board	21st Dynasty, Middle	Thebes (?)
"Museo Egizio" di Firenze	2157	Anonymous	Inner Lid	21st Dynasty, Middle	Thebes (?)
"Museo Egizio" di Firenze	7450	Pashuemipet	Inner Lid	21st Dynasty, Early	Thebes (?)
"Museo Egizio" di Firenze	9476	Tauhenut	Mummy Board	21st Dynasty, First half	Bab el-Gasus Cache
Egyptian Museum, Cairo	CG 6128	Anonymous	Mummy Board	21st Dynasty, Middle	Bab el-Gasus Cache
Egyptian Museum, Cairo	CG 6146	Anonymous	Outer Lid	21st Dynasty, Middle	Bab el-Gasus Cache
Egyptian Museum, Cairo	CG 6010	Pashedkhonsu	Outer Lid	21st Dynasty, Middle/ Late	Bab el-Gasus Cache
Fondazione Museo delle Antichità Egizie di Torino	Cat. 2222	Bakenkhonsu	Inner Lid	21st Dynasty, Late	Thebes (?)
Fondazione Museo delle Antichità Egizie di Torino	Cat. 2237/01	Butehamon	Inner Lid	21st Dynasty, Middle	Deir el-Medina (TT 291?)
Fondazione Museo delle Antichità Egizie di Torino	Cat. 223/03	Butehamon	Mummy Board	21st Dynasty, Middle	Deir el-Medina (TT 291?)
Fondazione Museo delle Antichità Egizie di Torino	Cat. 2236/01	Butehamon	Outer Lid	21st Dynasty, Middle	Deir el-Medina (TT 291?)
Fondazione Museo delle Antichità Egizie di Torino	Cat. 2212	Hori	Inner Lid	21st Dynasty, Middle	Thebes (?)
Fondazione Museo delle Antichità Egizie di Torino	Cat. 2212/01	Hori	Mummy Board	21st Dynasty, Middle	Thebes (?)
Fondazione Museo delle Antichità Egizie di Torino	S 7715/01	Horpaenaset	Inner Lid	21st Dynasty, Middle	Deir el-Medina
Fondazione Museo delle Antichità Egizie di Torino	Cat. 2238/01	Khonsumes	Inner Lid	21st Dynasty, Middle	Thebes (?)
Fondazione Museo delle Antichità Egizie di Torino	Cat. 2238/03	Khonsumes	Mummy Board	21st Dynasty, Middle	Thebes (?)
Fondazione Museo delle Antichità Egizie di Torino	S 7715/02	Mutemperamun	Mummy Board	21st Dynasty, Middle	Deir el-Medina
Fondazione Museo delle Antichità Egizie di Torino	Cat. 2219/01	Anonymous	Inner Lid	21st Dynasty, Late	Thebes (?)
Fondazione Museo delle Antichità Egizie di Torino	Cat. 2219	Anonymous	Mummy Board	21st Dynasty, Late	Thebes (?)
Fondazione Museo delle Antichità Egizie di Torino	Cat. 2226/02	Tabakenkhonsu	Inner Lid	21st Dynasty, Middle	Thebes (?)
Fondazione Museo delle Antichità Egizie di Torino	Cat. 2226/01	Tabakenkhonsu	Mummy Board	21st Dynasty, Middle	Thebes (?)
Fondazione Museo delle Antichità Egizie di Torino	Cat. 2228/01	Tamutmutef	Inner Lid	21st Dynasty, Late	Thebes (?)
Fondazione Museo delle Antichità Egizie di Torino	Cat. 2228/02	Tamutmutef	Mummy Board	21st Dynasty, Late	Thebes (?)
Los Angeles County Museum of Art	M.41.3a	Anonymous	Inner Lid	21st Dynasty, Middle	Thebes (?)
Los Angeles County Museum of Art	M.41.3c)	Anonymous	Mummy Board	21st Dynasty, Middle	Thebes (?)
Metropolitan Museum of Art, New York	17.2.7b.1	Amenhotep	Inner Lid	22nd Dynasty, Early	Sheikh Abd el-Gurna
Metropolitan Museum of Art, New York	17.2.7a.1	Amenhotep	Outer Lid	22nd Dynasty, Early	Sheikh Abd el-Gurna
Metropolitan Museum of Art, New York	26.3.4a	Ansenmes	Inner Lid	20th- 21st Dynasty	Deir el-Bahri (Pit 219)
Metropolitan Museum of Art, New York	26.3.8	Gautsoshen	Mummy Board	21st Dynasty, Late	Thebes (TT 60)
Metropolitan Museum of Art, New York	25.3.183a	Henuttawy	Inner Lid	21st Dynasty, Middle	Thebes (TT 59)
Metropolitan Museum of Art, New York	25.3.184	Henuttawy	Mummy Board	21st Dynasty, Middle	Thebes (TT 59)
Metropolitan Museum of Art, New York	25.3.182a	Henuttawy	Outer Lid	21st Dynasty, Middle	Thebes (TT 59)
Metropolitan Museum of Art, New York	86.1.5a	Iineferty	Inner Lid	19th Dynasty	Deir el-Medina (TT 1)
Metropolitan Museum of Art, New York	86.1.5c	Iineferty	Mummy Board	19th Dynasty	Deir el-Medina (TT 1)
Metropolitan Museum of Art, New York	26.3.1a	Iotefamun	Outer Lid	21st Dynasty, Early	Thebes (Pit 1016)
Metropolitan Museum of Art, New York	86.1.2a	Khonsu	Inner Lid	19th Dynasty	Deir el-Medina (TT 1)
Metropolitan Museum of Art, New York	86.1.1a	Khonsu	Outer Lid	19th Dynasty	Deir el-Medina (TT 1)
Metropolitan Museum of Art, New York	25.3.8a	Menkheperra	Inner Lid	21st Dynasty, Middle	Thebes (TT 60)
Metropolitan Museum of Art, New York	25.3.9	Menkheperra	Mummy Board	21st Dynasty, Middle	Thebes (TT 60)
Metropolitan Museum of Art, New York	25.3.7a	Menkheperra	Outer Lid	21st Dynasty, Middle	Thebes (TT 60)
Metropolitan Museum of Art, New York	25.3.11a	Tabakmut	Inner Lid	21st Dynasty, Middle/ Late	Thebes (TT 60)
Metropolitan Museum of Art, New York	25.3.12	Tabakmut	Mummy Board	21st Dynasty, Middle/ Late	Thebes (TT 60)
Metropolitan Museum of Art, New York	25.3.10a	Tabakmut	Outer Lid	21st Dynasty, Middle/ Late	Thebes (TT 60)
Metropolitan Museum of Art, New York	25.3.15a	Tiye	Inner Lid	21st Dynasty, Middle	Thebes (TT 60)
Metropolitan Museum of Art, New York	25.3.16	Tiye	Mummy Board	21st Dynasty, Middle	Thebes (TT 60)
Musée du Louvre, Paris	AF 9592	Aafenhor	Inner Lid	21st Dynasty, Middle	Akhmim (?)
Musée du Louvre, Paris	E 13030	Amenhotep	Inner Lid	21st Dynasty, Late	Thebes (?)
Musée du Louvre, Paris	E 13041	Amenhotep	Mummy Board	21st Dynasty, Late	Thebes (?)
Musée du Louvre, Paris	E 13028	Amenhotep	Outer Lid	21st Dynasty, Late	Thebes (?)
Musée du Louvre, Paris	E 27460	Bakenmut	Inner Lid	22nd Dynasty	Thebes (?)
Musée du Louvre, Paris	E 13047	Nebhep	Mummy Board	21st Dynasty, Early	Deir el-Medina (?)
Musée du Louvre, Paris	AF 9590	Anonymous	Inner Lid	21st-22nd Dynasty	Thebes (?)
Musée du Louvre, Paris	E 10636	Anonymous	Inner Lid	21st Dynasty, Late	Bab el-Gasus Cache
Musée du Louvre, Paris	E 10636	Anonymous	Outer Lid	21st Dynasty, Late	Bab el-Gasus Cache
Musée du Louvre, Paris	E 13036	Anonymous	Inner Lid	21st Dynasty, Middle	Thebes (?)
Musée du Louvre, Paris	E 13045	Anonymous	Inner Lid	21st-22nd Dynasty	Thebes (?)
Musée du Louvre, Paris	18840	Anonymous	Inner Lid	21st-22nd Dynasty	Thebes (?)
Musée du Louvre, Paris	18840	Mererimenna	Mummy Board	21st-22nd Dynasty	Thebes (?)
Musée du Louvre, Paris	E 3859	Anonymous	Mummy Board	21st Dynasty, Middle	Thebes (?)
Musée du Louvre, Paris	E 13029	Panebmonthu	Inner Lid	21st Dynasty, Early	Thebes (?)
Musée du Louvre, Paris	E 13046	Panebmonthu	Mummy Board	21st Dynasty, Early	Thebes (?)
Musée du Louvre, Paris	E 20165	Paser	Mummy Board	21st Dynasty, Early	Thebes (?)
Musée du Louvre, Paris	N 2581	Paser	Outer Lid	21st Dynasty, Early	Thebes (?)
Musée du Louvre, Paris	N 2610	Sutymes	Inner Lid	21st Dynasty, Early	Deir el-Medina (?)
Musée du Louvre, Paris	N 2611	Sutymes	Mummy Board	21st Dynasty, Early	Deir el-Medina (?)
Musée du Louvre, Paris	N 2609	Sutymes	Outer Lid	21st Dynasty, Early	Deir el-Medina (?)
Musée du Louvre, Paris	N 2571	Tamutneferet	Inner Lid	19th Dynasty	Sheikh Abd el-Gurna
Musée du Louvre, Paris	N 2620	Tamutneferet	Mask	19th Dynasty	Sheikh Abd el-Gurna
Musée du Louvre, Paris	N 673	Tamutneferet	Outer Lid	19th Dynasty	Sheikh Abd el-Gurna
Musée du Louvre, Paris	E 13035	Tanethereret	Mummy Board	21st Dynasty, Early	Thebes (?)
Musée du Louvre, Paris	E13034	Tanethereret	Inner Lid	21st Dynasty, Early	Thebes (?)
Musée du Louvre, Paris	E13027	Tanethereret	Outer Lid	21st Dynasty, Early	Thebes (?)
Musée du Louvre, Paris	N 2562	Tanetimen	Inner Lid	21st Dynasty, Middle	Deir el-Medina (?)
Musée du Louvre, Paris	N 2612	Tanetshedmut	Inner Lid	22nd Dynasty, Early	Thebes (?)
Musée du Louvre, Paris	E 18843	Tchanefer	Inner Lid	21st Dynasty, Middle	Thebes (?)
Musei Vaticani, Città del Vaticano	25003.2.1	Amenhotep	Inner Lid	21st-22nd Dynasty	Thebes (?)
Musei Vaticani, Città del Vaticano	D 2066.2.1	Anet	Inner Lid	20th- 21st Dynasty	Deir el-Medina/ Akhmim (?)
Musei Vaticani, Città del Vaticano	25012.2.1	Djedhoriuefankh	Outer Lid	22nd Dynasty, Early	Thebes (?)
Musei Vaticani, Città del Vaticano	25008.2.1	Djedmut	Outer Lid	21st-22nd Dynasty	Thebes (?)
Musei Vaticani, Città del Vaticano	25035.3.2	Ikhy	Mummy Board	21st Dynasty, Middle/ Late	Bab el-Gasus Cache
Musei Vaticani, Città del Vaticano	25035.3.1	Ikhy	Outer Lid	21st Dynasty, Middle/ Late	Bab el-Gasus Cache
Musei Vaticani, Città del Vaticano	25016.2.1	Anonymous	Inner Lid	21st Dynasty, Middle/ Late	Bab el-Gasus Cache
Musei Vaticani, Città del Vaticano	51515	Anonymous	Inner Lid	21st Dynasty, Late	Bab el-Gasus Cache
Musei Vaticani, Città del Vaticano	25020	Anonymous	Mummy Board	21st Dynasty, Late	Bab el-Gasus Cache
Musei Vaticani, Città del Vaticano	25022	Anonymous	Mummy Board	21st Dynasty, Late	Bab el-Gasus Cache
Musei Vaticani, Città del Vaticano	25015.2.1	Takhybiat	Inner Lid	21st Dynasty, Late	Bab el-Gasus Cache
Museo Archeologico Nazionale di Napoli	2348	Nesra	Inner Lid	22nd Dynasty, Early	Thebes (?)
Museo Archeologico Nazionale di Napoli	2347	Anonymous	Inner Lid	21st-22nd Dynasty	Thebes (?)
National Museum of Egyptian Civilization	CG 61011	Padiamon	Inner Lid	19th-20th Dynasty	Royal Cache (TT 320)
Rijksmuseum van Oudheden, Leiden	AMM18-g	Ankhefenkhonsu	Outer Lid	22nd Dynasty, Early	Thebes (?)
Rijksmuseum van Oudheden, Leiden	AMM18-h	Djedmonthuiufankh	Inner Lid	22nd Dynasty, Early	Thebes (?)
Rijksmuseum van Oudheden, Leiden	AH1a	Nesypanebawib	Mummy Board	21st Dynasty, Early	Thebes (?)
Rijksmuseum van Oudheden, Leiden	F 93/10.2b.1	Nesytanebtawy	Inner Lid	21st Dynasty, Late	Bab el-Gasus Cache
Rijksmuseum van Oudheden, Leiden	F 93/10.2a.1	Nesytanebtawy	Outer Lid	21st Dynasty, Late	Bab el-Gasus Cache
Rijksmuseum van Oudheden, Leiden	L1	Anonymous	Inner Lid	21st-22nd Dynasty	Thebes (?)
Rijksmuseum van Oudheden, Leiden	F 93/10.4.1	Anonymous	Inner Lid	21st Dynasty, Late	Bab el-Gasus Cache
Rijksmuseum van Oudheden, Leiden	F 19/31.9.1	Anonymous	Inner Lid	21st Dynasty	Thebes (?)
Rijksmuseum van Oudheden, Leiden	AH 188	Penpy	Mummy Board	22nd Dynasty, Early	Thebes (?)
Rijksmuseum van Oudheden, Leiden	F 93/10.3a	Tentpenherunefer	Inner Lid	21st Dynasty, Late	Bab el-Gasus Cache
Rijksmuseum van Oudheden, Leiden	F 93/10.3b	Tentpenherunefer	Mummy Board	21st Dynasty, Late	Bab el-Gasus Cache

The *corpus* represents a heterogeneous group of objects for provenance, chronology and typology. For the most part we have no secure geographical provenance since they were acquired by private collection at the beginning of the 19 ^th^ century. Beyond these, are also objects from the Bab el-Gasus Cache, the collective tomb discovered in 1891 close to the temple of Hatshepsut with the burials of 153 priests and priestesses of Amun who lived under the 21st Dynasty (ca. 1069–945 BC) ^23^. In this research are included the coffins of the Lot I, stored in the Musée du Louvre in Paris; the Lot V in the “Museo Egizio” di Firenze; the Lot XI in the Rijksmuseum van Oudheden (RMO) in Leiden; the Lot XVII in the Musei Vaticani and the outer coffin of Pashedkhonsu (CG6010) and the anonymous outer coffin with related mummy board (CG6146, CG6128) stored in the Egyptian Museum in Cairo (EMC) ^24^. In addition to the Bab el-Gasus Cache, the *corpus* includes coffins from other areas of the Theban Necropolis such as the Royal Cache ^25^ and tombs in Deir el-Medina.

In this research all the types and subtypes identified by Niwinski in 1988 are considered, covering then the entire period of production/ use of yellow coffins: from the “first well–authenticated examples of coffins of yellow type” ^26^ in the 19 ^th^ dynasty - the so called proto yellow coffins from the Tomb of Sennedjem (TT1) ^27^ - until the beginning of the 22 ^nd^ dynasty with the *stola* coffins ^28^.

### Photogrammetric protocol and data processing

The aim of the Faces Revealed Project is the construction of high-resolution and submillimetric 3D representations of coffins capable of describing every fine detail with and without texture by means of versatile, transportable and cost-effective instruments and software. For these reasons the photogrammetric approach appeared to be more useful for this project in comparison with a 3D scanner. Even the accuracy of the photogrammetric 3D models depends on many different factors, these can be taken under control and the models can have comparable resolution than the scanners ^29^ or, at least, meet the needs of the Faces Revealed Project.

In collaboration with the Department of Architecture, Built Environment and Construction engineering (DABC) of the Politecnico di Milano ^30^, the first operation was to find the best instruments and methodology ^31^. Then, a specific reference protocol for 3D acquisition was developed. The protocol included the possibility of adaptions for different contexts according to museums’ conditions.

Faces Revealed Project considers only the external upper part of coffin lids as far down as the lower part of the crossed forearms or, for the *stola* coffins, until the end of the collar ( *wesekh*-collar). The coffins were placed in a horizontal position (on the floor or on a table). A full-frame Nikon D750 camera coupled with a Nikkor 35 mm f/1.8 lens was used for the photographic survey. A polariser was mounted on the camera’s lens to avoid as much as possible reflections and shiny areas due to the varnish on the coffins. The yellow coffins are in fact covered by an original ( *Pistacia* resin) or modern (Paraloid B72) ^32^ layer of varnish. Ancient or modern, the varnish creates reflections, which could produce a layer of noise and subsequent blunders and incorrect descriptions of the coffin’s shape ^33^. This issue can be overcome using the polariser ^34^, which became an essential instrument of the project (see *infra*).

Each image has a maximum resolution of 6016 pixels by 4016 pixels. The camera is always placed on a tripod and the shot is delayed by 5 seconds - using automatic shutter release or a remote control - to prevent vibration. This permits the use of low ISO values (no more than 800) in the low-illuminated museum rooms. The mean calculated Ground Sampling Distance (GSD) of the photogrammetric survey is around 0,2 mm since the pixel pitch is 6 μm and the distance of acquisition from the object is always about 1 m. The acquisition geometry is circular around the object, maintaining the camera as much as possible nadiral to the coffins. The number of images for each artefact is variable considering both the dimension of the objects and a transversal overlap of the images of more than 85% (between 90–150 pics for half coffins until 300 for a full coffin).

**Figure 1.**
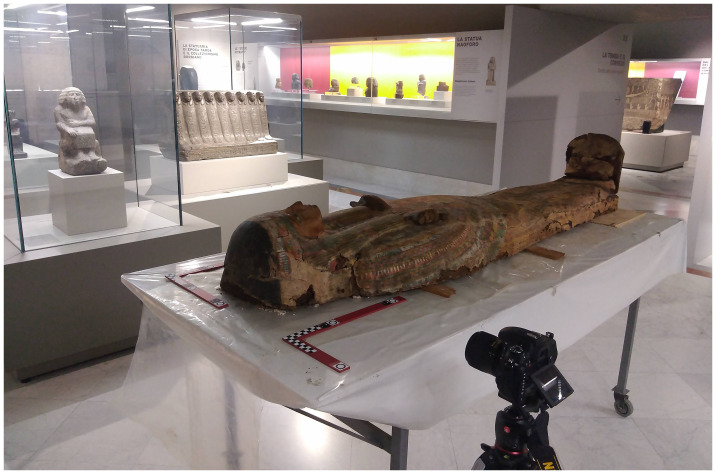
Photogrammetric survey of the lid of Nesra (MANN, inv. n. 2348). The image is courtesy and authorised by the Museo Archeologico Nazionale di Napoli (MANN).

Other essential instruments for the photogrammetric survey are two pre-calibrated L-shaped bars. The bars are iron carpenter’s squares of 60 and 40 cm, always placed next to the head of each coffin and on the same level ( Figure 1). The bars have been equipped with circle and cross non-coded targets fundamental to prevent deformations in the final 3D model. Moreover, these are useful to accurately scale the reconstruction accurate to 0.001(m) and to have a precise reference system that defines the plane on which the orthophotos are then projected.

As mentioned, on the basis of the different environments, methods and techniques of data acquisition were adapted at times. The main variables addressed during the survey were linked to: i) the low illumination of the exhibition rooms, ii) the inability to open the display case in which the objects are kept, iii) and the position of the coffins.

For dark rooms two different solutions were applied linked to the use or not of the flash (Yongnuo YN560). A flash synchronised with the camera was used for the inner lid and mummy board of Butehamon in the Museo Egizio, Torino (Cat. 2237/01 and Cat. 2237/03) ^35^. Even if the objects were placed outside the showcases and in a horizontal position, the survey was made in the Museum’s exhibition room n. 8, a dark area with little and yellow light from the ceiling. Moreover, the colour of the coffins is very dark, and the objects are covered by varnish, creating accentuated reflective surfaces. To reduce the noises, the polariser was used with constant control, image by image, and a manual adjustment, as well as a long and elaborated transformation of pictures by Lightroom were performed.

**Figure 2.**
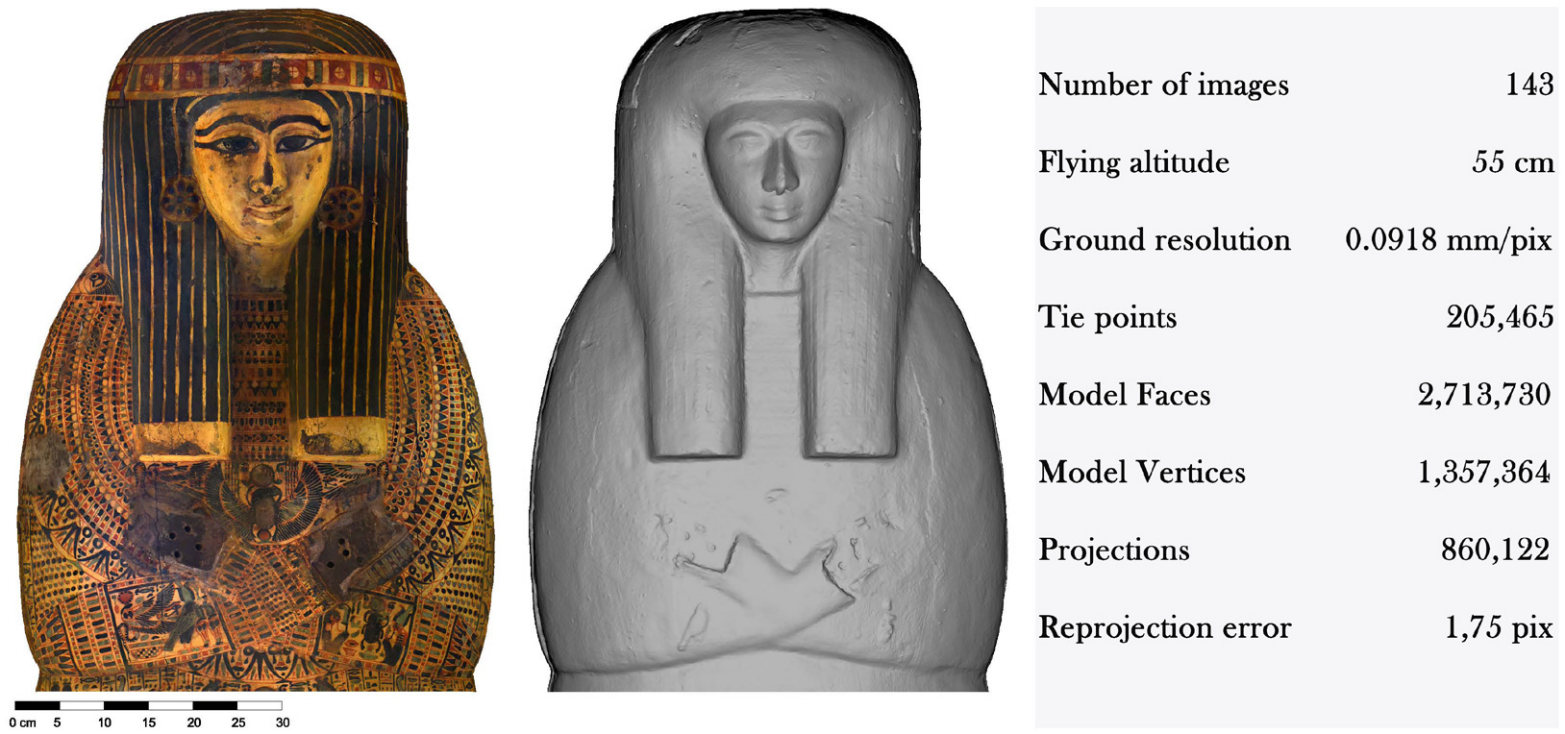
Textured and non textured orthophoto of the lid of Khonsumes (“Museo Egizio”, Firenze, inv. n. 8527). The orthophotos are courtesy and authorised by the Museo Archeologico Nazionale di Firenze (Direzione Regionale Musei della Toscana).

The second solution adopted was to use the camera mounted on the tripod without flash, using a remote control and a small light switched on only for the time to focus the framed area before releasing the shutter. This setting was the best solution for coffins in dark rooms and for those coffins that are closed in showcases. Taking pictures through the display windows represented the most challenging situation during the present research. Even if the work is quite long both for the survey and for the post processing, the results are functional both in terms of resolution and error ( Figure 2).

**Figure 3.**
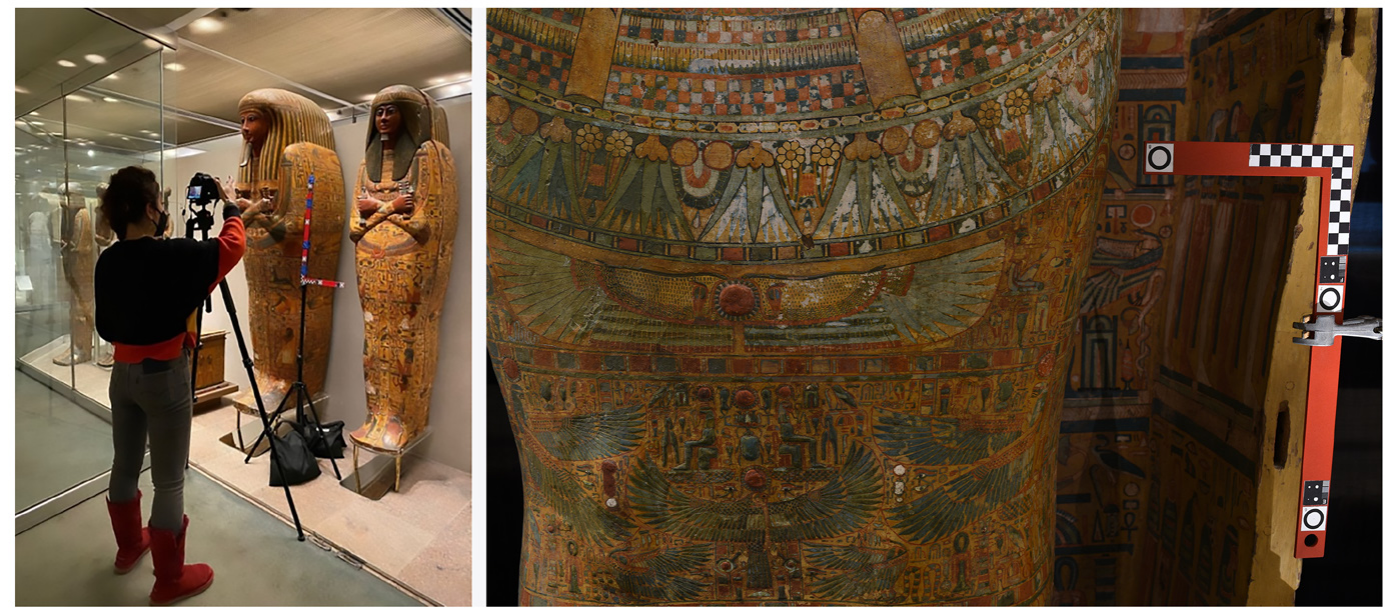
Photogrammetric survey of the lid of Khonsu (MET, 86.1.2a) and of Ankhefenkhonsu (RMO, AMM18-g1). The images are courtesy and authorised by the Metropolitan Museum of Art, New York and the Rijksmuseum van Oudheden, Leiden.

The protocol developed also had to be adapted when the coffins were placed in a vertical position, and it was not possible to move them from their casing. In this case, the bars were fixed on a tripod using tweezers and placed as close as possible to the object, on one side or in front of it ( Figure 3). On one hand this position made it easier to place the camera nadiral to the coffin; on the other hand, due to the narrow spaces, it was not always possible to reach the top and the sides of the object, so sometimes the 3D models lack these portions.

The 3D photogrammetric data elaboration uses the software Agisoft Metashape Professional 1.8.3. and strictly follows the photogrammetric pipeline: i) photo-alignment at high resolution; ii) automatic recognition and manual check of circular and cross non-coded targets; iii) scaling; iv) dense cloud reconstruction in “medium” or “high” quality and data cleaning; v) mesh model in “medium” or “high” quality; vi) texturing; developing high-resolution orthophoto; vii) exporting high-resolution orthophoto textured and non textured ^36^.

### The “construction” of different layers. Comparison, overlapping and results

The 3D models are fundamental since they represent the only way to export identical and high-resolution images of an object, both textured and non textured. These images represent the first two “layers” created for each coffin: the Visual appearance “layer” (the orthophoto with texture – “layer” 1) and the Physical geometry “layer” (the orthophoto without texture – “layer” 2). The extraction of the model in both cases follows the coordinate and the metric system given during the photographic survey ^37^. The two models exported have an identical orthogonal projection and can be accurately overlapped ^38^.

**Figure 4.**
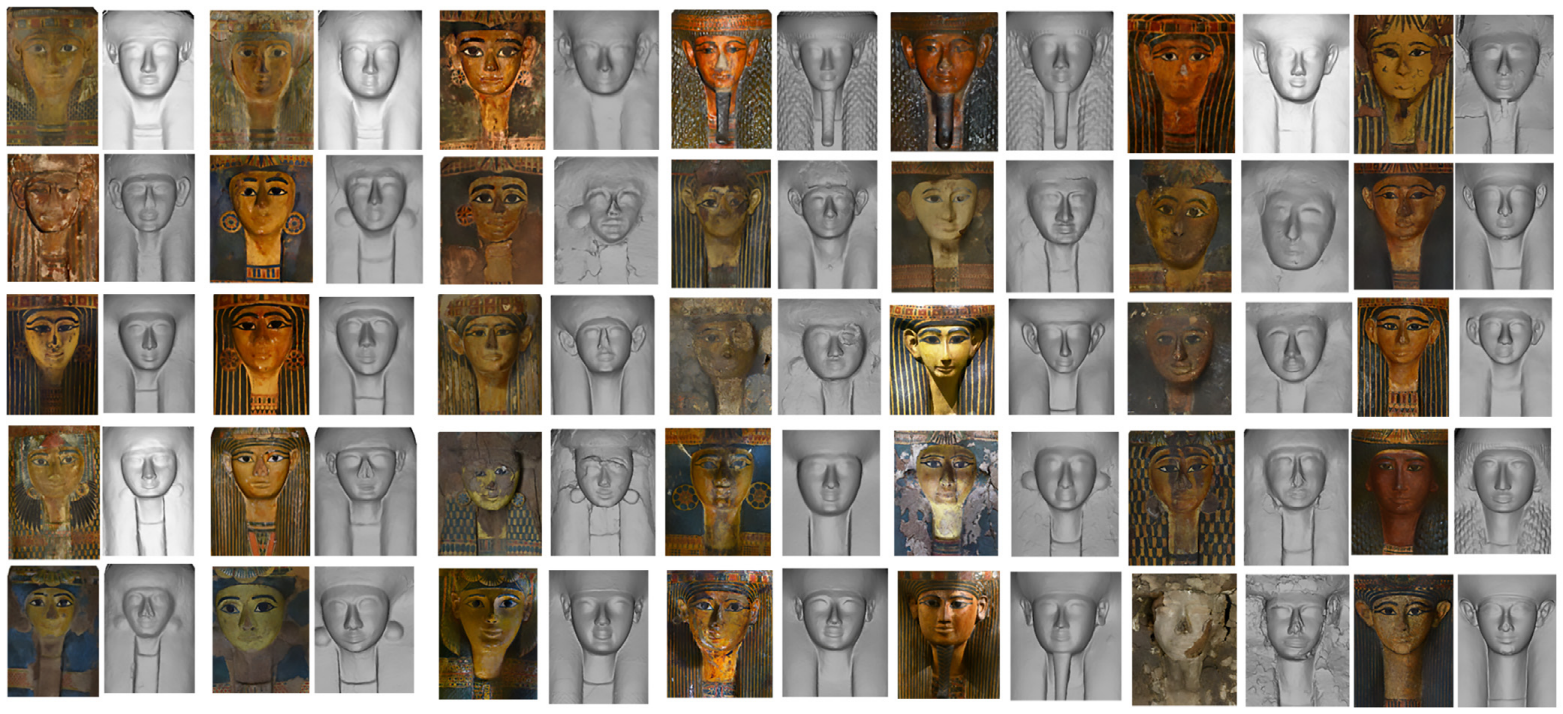
Textured and non textured faces of yellow coffins. The images are courtesy and authorised by the Museums Partner: Museo Archeologico Nazionale di Napoli (MANN); Museo Archeologico Nazionale di Firenze (Direzione Regionale Musei della Toscana); Rijksmuseum van Oudheden, Leiden; Musée du Louvre, Département des Antiquités égyptiennes; Vatican Coffin Project, Governatorato S.C.V. - Direzione dei Musei; Museo Egizio di Torino.

The “layers” allow us to have the first autoptic inspection of the difference between lids with and without colour. When the decoration layer (“layer” 1) is switched off it is possible to better observe some peculiarities and particulars that were indiscernible to the naked eye due to the paint covering. Comparing the two visualisations it is clear that in most cases the objects or some of their elements can appear very different in their geometry and show variability. The faces, for example, are not idealised faces, serially produced with schematic and homogeneous facial features, but all of them present specific traits with different forms, positions and dimensions of the oval, eyes, eyebrows, nose, mouth, ears and earrings ( Figure 4).

**Figure 5.**
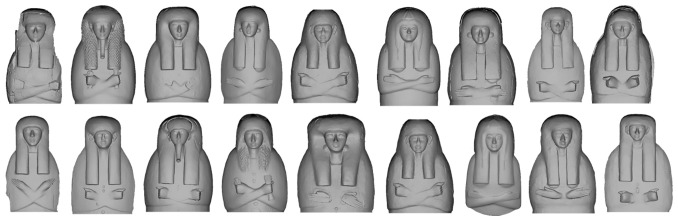
Variability in the rendering of the forearms in non textured orthophotos. The orthophotos are courtesy and authorised by Museo Archeologico Nazionale di Firenze (Direzione Regionale Musei della Toscana); Rijksmuseum van Oudheden, Leiden; Musée du Louvre, Département des Antiquités égyptiennes; Vatican Coffin Project, Governatorato S.C.V. - Direzione dei Musei; Museo Egizio di Torino; National Museum of Egyptian Civilization (NMEC), Cairo; Egyptian Museum, Cairo.

This huge variability in the forms and geometry, especially compared to the use of paint, regards not only the masks, but also the wigs, the hands and is particularly evident in the rendering of forearms. In painting we only have 3 ways to draw the forearms: arms and forearms fully visible (Type I), partially covered by a large collar (Type II) and fully covered by the collar (Type III) ^39^. These three different representations of the forearms were considered a key index for the typology of yellow coffin in terms of a chronological evolution: from oldest to newest ^40^. However, geometrically, scholars only considered if arms and forearms were fully modelled or missing ^41^. Nevertheless, “layer” 2 shows that there is much greater variability in the rendering of forearms with huge degrees of variation from one coffin to another starting from the full or partial representation of forearms to their orientation. The forearms, in fact, can be fully or partially represented, they can be arranged transversally or in a V-form, they can have merely the elbow rendered or nothing at all, they can have just a squared or rounded lower line, the cross can be fully rendered or not at all and can even be wrong - with the left forearm placed upon the right one ( Figure 5).

**Figure 6.**
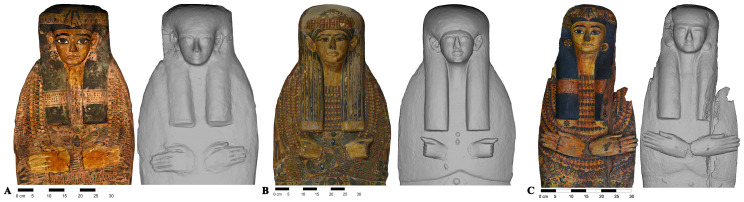
**a**- **c** Comparison between textured and non textured orthophotos of three mummy boards. The orthophotos are courtesy and authorised by Museo Archeologico Nazionale di Firenze (Direzione Regionale Musei della Toscana); Rijksmuseum van Oudheden, Leiden; Museo Egizio di Torino.

Moreover, when forearms are represented in decoration, it cannot be automatically predicted that they are also rendered in the geometry. In fact, sometimes the forearms are only painted on a flat surface (i.e. the mummy board of Ankhsenmut, “Museo Egizio” di Firenze, inv. n. 8521, Figure 6a), they are fully rendered in the decoration but are marked by a squared/rounded lower line to create light volume and three-dimensionality, even if the surface is completely flat (i.e. the mummy board of Nesypanebawib, RMO Leiden, AH 1a, Figure 6b). In contrast we can sometimes detect proof of the original forearms in the geometry which are completely concealed under a large painted collar (i.e. the mummy board of Mutemperamun, Museo Egizio, Torino, S. 7715/02, Figure 6c).

So far, the comparison between the Visual appearance and Physical geometry has yielded fascinating and unexpected results especially for those full coffin sets that are formed by an outer coffin, an inner coffin, and a mummy board. In terms of Visual appearance, there are many cases where the figural decoration, the layout of that decoration, as well as the palette and the style used for all the pieces are clearly the same and applied by the “same hand”. By switching the colour off we face one of two different situations: either we can see the same strong links also in the Physical geometry or we see that such links existing in the decoration do not exist in the geometry and that the different pieces forming the same set are actually very different from each other in their Physical geometry. The two possibilities are clearly visible, for example, in the coffin set of Amenhotep in the Musée du Louvre, Paris (Louvre, inv. nos. E 13028, E13030, E 13041) and in the set of Tabakmut in the Metropolitan Museum of Art in New York (MET inv. nos. 25.3.10a, 25.3.11a, 25.3.12).

**Figure 7.**
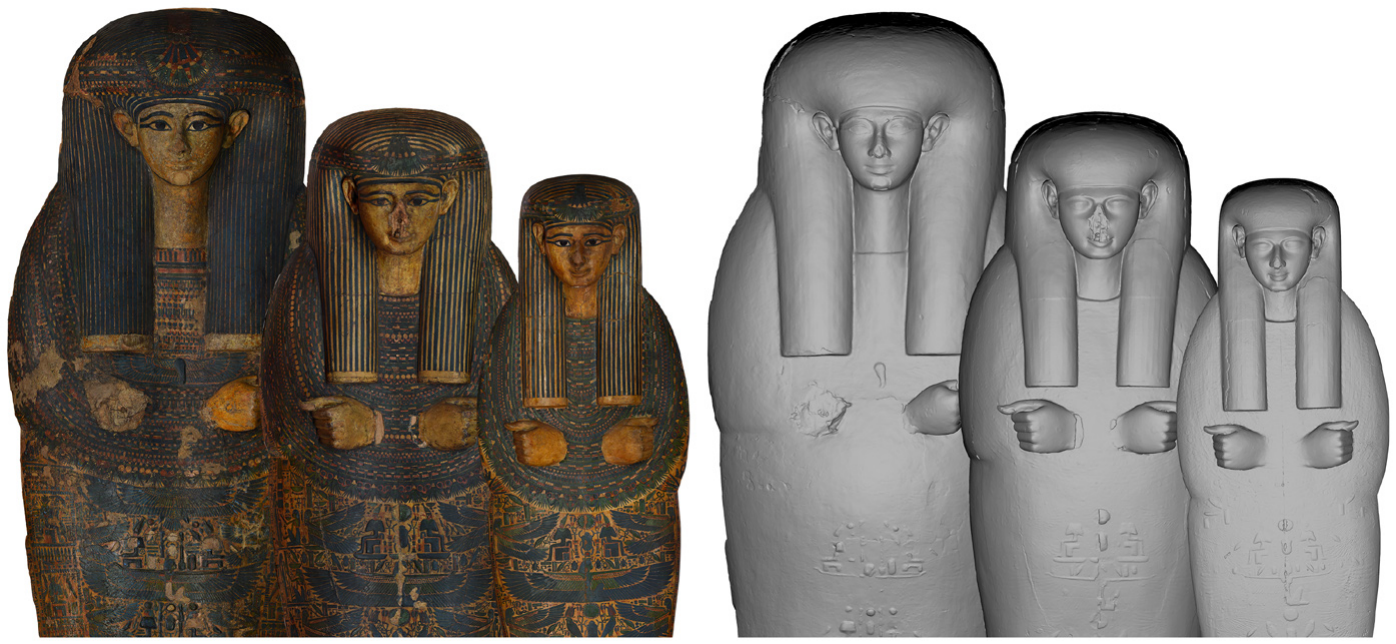
Textured and non textured orthophotos of the coffin set of Amenhotep (Musée du Louvre, Paris, inv. nos. E 13028, E13030, E 13041). The orthophotos are courtesy and authorised by the Musée du Louvre, Département des Antiquités égyptiennes.

The set of the *wab*-priest Amenhotep in Louvre was presented by F. Cailliaud to the Bibliothèque Nationale in 1825 and likely comes from Thebes ( Figure 7) ^42^.

The set was dated to the late 21st Dynasty on the basis of the decoration and layout (types IIIb and IIIc), which follows the same conventions in all three pieces. The masks are framed by striped wigs with two or three bands of headbands and a bunch of lotus flowers hanging down from the crown to the head and horizontal end yellow bands. The large collar partially covers the forearms on all the objects and only the elbows are drawn with a lotus flower (type II). The hands, crossed and closed as usual in coffins for male deceased, are attached to the chest. The colour palette is above all dark green and blue on a yellow background and some elements were formed by moulded plaster applied as raised relief. The same uniformity in decoration can also be seen in the geometry, especially on the inner lid and the mummy board: the faces have a short and large forehead with a marked line of the wig which ends on the temples into two short edges in relief. The faces are squared with rounded eyebrows and almond-shaped eyes with a marked shade of circles in the internal part, smiling mouths with thin lips in a particular V-form and protruding and squared chins. The connection in the carving of the faces, the hands and the ears suggest the same craft production brings us to the conclusion that the two pieces were produced to match each other both in craft and in decoration.

**Figure 8.**
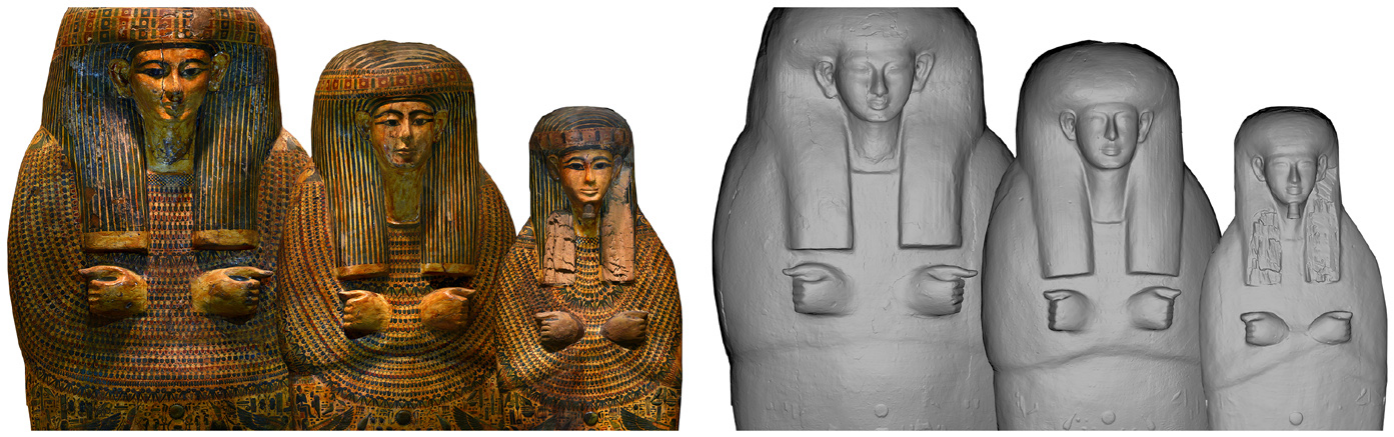
Textured and non textured orthophotos of the coffin set of Tabakmut (MET, inv. nos. 25.3.10a, 25.3.11a and 25.3.12). The orthophotos are courtesy and authorised by the Metropolitan Museum of Art, New York (MET).

The coffin set of Tabakmut in the MET, shows a completely different situation. The set was found in chamber 2 of Theban Tomb n. 60 (TT 60) during the MMA excavations of 1923–24 ^43^ ( Figure 8).

The three pieces have a great uniformity in decoration and layout and also in this example the style of the decoration suggests the same “hand”, especially in the characteristic and not so common blue/grey paint in the hand hollows on all three objects found – until now - only on one other coffin ^44^. On all the pieces the deceased wears a tripartite wig with one or three lines of the crown of justification and a broad collar with falcon heads on the shoulders which cover the forearms. Even if the decoration is the same (type IIIa) ^45^ and dated to the Middle-Late 21 ^st^ Dynasty, they are completely different in their Physical geometry. In the Visual appearance, for example, the forearms are only partially painted (type II) but they are also modelled and clearly visible under the collar on all three pieces. In the outer and inner lids, they are simply realised with curved lines arranged transversally, while in the mummy board there is the full rendering of the crossing with the right arm on the left one.

**Figure 9.**
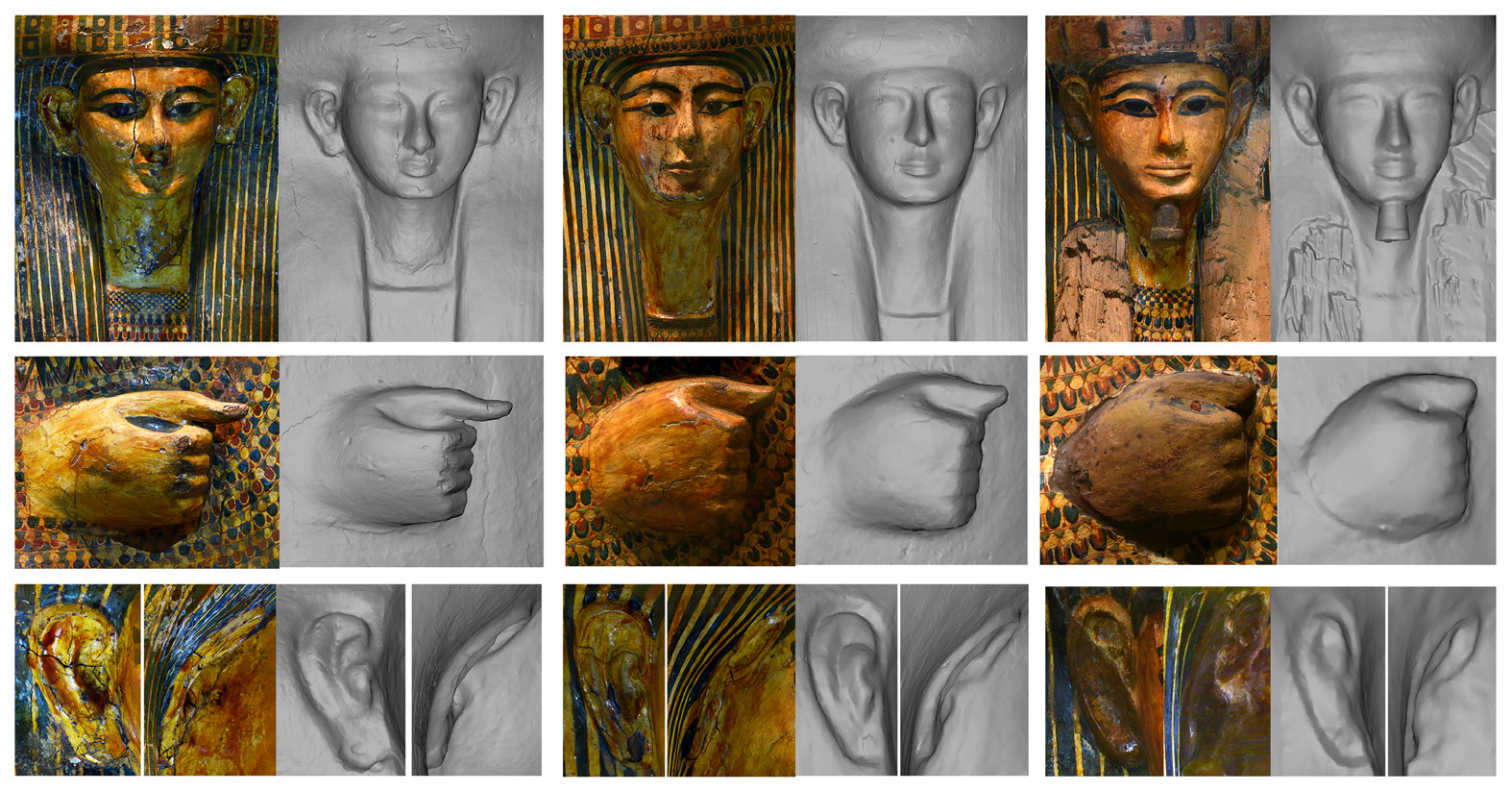
Particular of the faces, hands and ears of the coffins of Tabakmut (MET, inv. nos. 25.3.10a, 25.3.11a and 25.3.12). The orthophotos are courtesy and authorised by the Metropolitan Museum of Art, New York (MET).

Beyond this macroscopic particularity, another characteristic of the set is that all the masks, ears and hands are very different to one other, and the outer and inner masks have very distinctive facial features ( Figure 9). 

After the autoptic comparison, the second phase of the methodology is the creation of another two “layers” for the objective analysis on how the decoration is applied on the three-dimensional features. The two layers have been created through the one open-source paint.net software ^46^. One “layer” is dedicated to tracing the decoration (considering only the elements which are objects of the study and not the full decoration – “layer” 3) while the other “layer” to highlight the geometry through points (“layer” 4) have been created ^47^. These two “layers” can be overlapped in transparency on the solid model, on the texturised model and also between them, thereby maintaining the same orientation and the same proportions. This image overlapping enables a more precise inspection of objects and tracing the conformity of the two layers giving an insight into how ancient artists applied the coloured features on the three-dimensional masks, analysing any possible corrections that were applied to the geometry through the paint.

The overlapping of the four “layers” demonstrates that only in 37% of the coffins does the Visual appearance and the Physical geometry match exactly. There was a 13% mismatch on at least three painted features while in 46% they partially match, i.e. less than 3 features. The mismatch can relate to the form of the mouth and/or the thickness and form of the lips, as well as the ears which can be reduced in dimension when painted. Similarly, the mismatch could be seen in the form of the face (both bigger or smaller), in the position of the painted eyebrows and eyes on the mask, in the form of the arms, even if the features that, in percentage terms, do not match are above all the eyes, ears, and mouth/lips.

**Figure 10.**
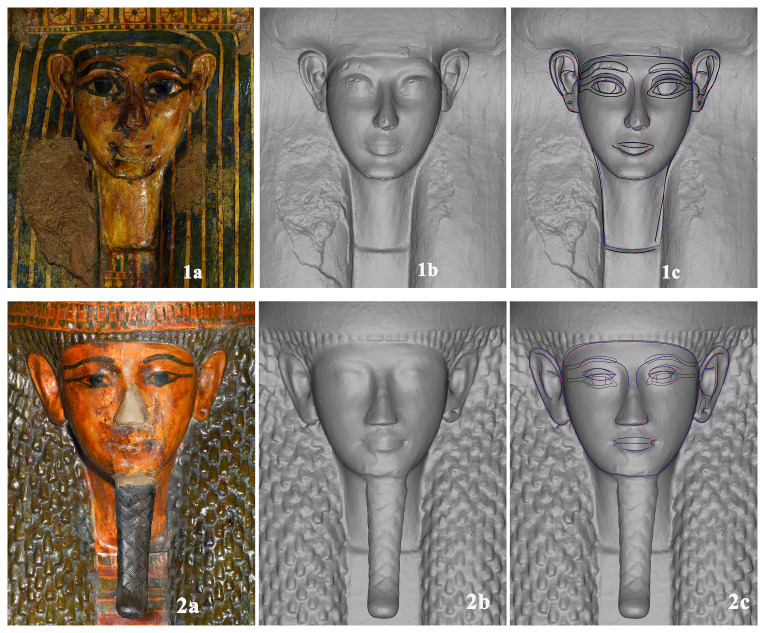
Particular of the matches between the “Layers”: Khonsumes ( **1a**– **c**) and Butehamon ( **2a**– **c**) (Museo Egizio, Torino, Cat. 2238/01 and 2237). The orthophotos are courtesy and authorised by the Museo Egizio, Torino.

When we say that the different layers exactly correspond to each other it means that the paint does not transform the original crafted features and that the decoration maintains the same proportions, features and forms realised in the geometry ( Figure 10, 1a–c). Comparing the two visualisations, then, we can recognise the object. The same can be observed when there is a partial match, with a modification of less than 3 features. The decoration, in fact, seems to modify only a few features linking the discrepancies between layers more to a specific production or to adjustment and correction of errors - such as asymmetry between the two sides of the face - more than a deliberate will to transform the mask ( Figure 10, 2a–c).

On the contrary, when we talk about full mismatch in decoration we have a completely different perception of the mask with and without decoration and, in this case, it is clear that colour was used to transform and modify the original crafted features. A very interesting example of changing forms and proportions of the mask through decoration is the outer lid of the Charioteer of the Overseer of the Army, Iotefamun (MET 26.3.1a) ^48^. The coffin, discovered in 1920–21 in pit 1016 at the Mentuhotep temple area in Deir el-Bahari ^49^, was found with an inner coffin (MET 26.3.2a–b) ^50^ and a mummy board (MET 26.3.3) ^51^ which are now in the Virginia Museum of Fine Art (VMFA, L.7.47.77) ^52^.

**Figure 11.**
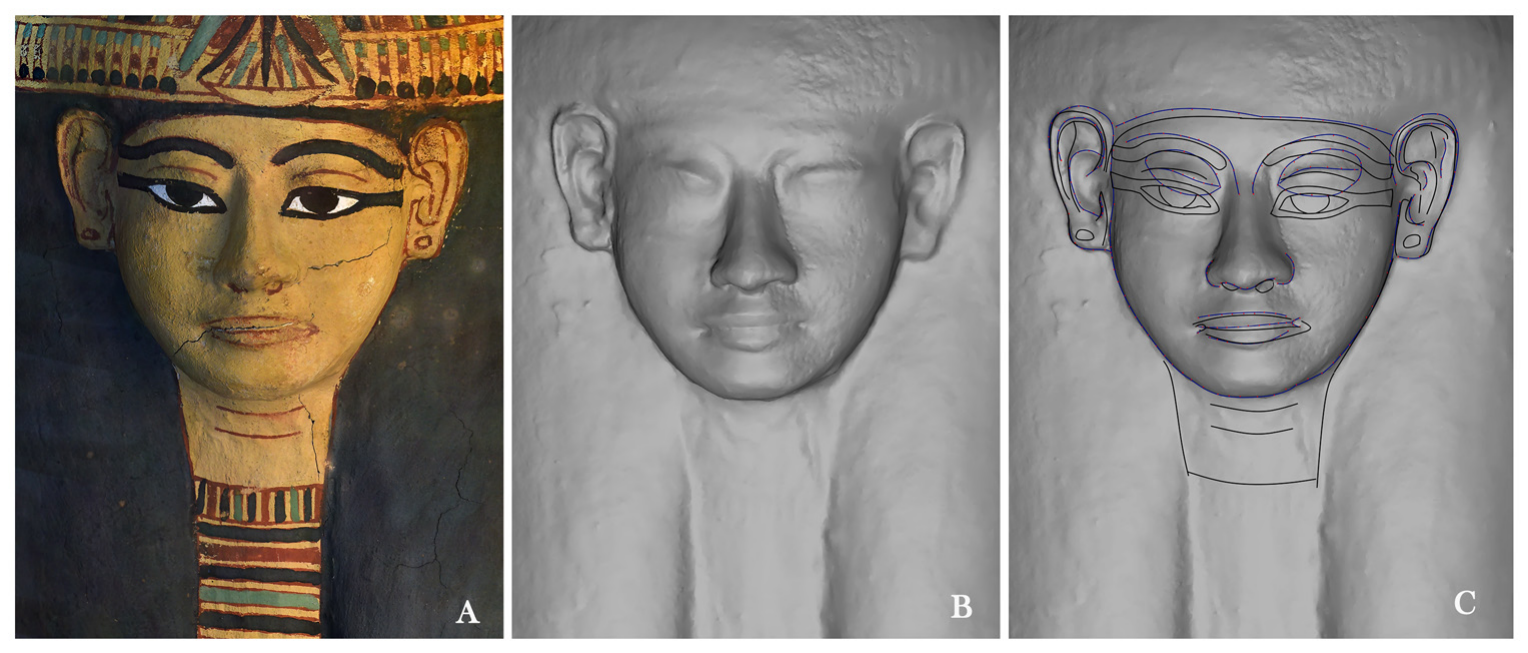
Particular of the matches between the “layers”: Iotefamun (MET, inv. n. 26.3.1a). The orthophotos are courtesy and authorised by the Metropolitan Museum of Art, New York (MET).

The outer lid of Iotefamun corresponds to the type IIa and was dated by Niwiński firstly at the early 21 ^st^ dynasty (1000 BCE ca.) ^53^ and later to the late Ramesside period (1295–1069 BCE ca.) ^54^. The lid represents a male deceased as testified by the closed hands and the uncovered ears (male gender markers). The face is drawn in a red line over the yellow ochre with black hieroglyphic eyes framed by curved eyebrows becoming straight on the side and aligned with the makeup, and with a thin red line traced between the eyes and the eyebrows to define the eyelids. The nose is large with nostrils in red and the mouth is unsmiling, schematic and large with thin lips and circles at the corners. The deceased wears a long tripartite plain blue wig, the ears are uncovered and naturalistic in style with lines and holes on the lobe in red ( Figure 11a).

On the geometric point of view the outer lid is rounded in shape with a protruding belly, crossed forearms carved in high relief with the right arm on the left one and close, small and squared hands with stubby thumbs. The wig is rounded with short lappets, but was originally curled. Geometrically, it is clear that the wig was intentionally modified and transformed from a curly to a plain one. The waves, hardly visible to the naked eye, are clearly recognisable in the geometry at the top and the outermost lateral sides. This flattening modification was executed in a rough manner using both a dark colour and a thin layer of plaster. The face is rounded with a high and flat forehead, high cheekbones, full cheeks and an oval chin; the forehead is flat and high but presents a line in the middle, as a kind of modification; The eyebrows are arched and frame two highly protruding, oblique, and almond-shape eyes located at the level of the root of the nose; the nose is big, narrow at the root and large at the base; the mouth is unsmiling and narrow with thin lips and hollows in the corners; the ears, naturalistic and big with separate lobe, are located in the area between the line of the wig and the base of the nose; the neck is three-dimensionally rendered but small and with any line of separation with the collar ( Figure 11b).

Even if it is already clear that the mask looks completely different without paint, overlapping the layers we can analyse in more detail these differences and see how the colour transformed the forms. In addition to the modification of the wig, the proportion and the facial features were clearly modified ( Figure 11c):

i. the oval of the face is reduced in the upper part colouring partially the forehead;
ii. the big ears are reduced covering the external part of the pavilion, an element found also in the coffin set of Butehamon ^55^;
iii. the black eyebrows do not follow the modelled arched form ^56^ but are drawn in the space between the line of the upper eyelid and the eyebrow;
iv. the eyes start under the bulged three-dimensional eyes and appear painted in the upper part of the cheeks;
v. the red line for the eyelid is in the wrong position, not in the curvature but painted upon the most prominent part of the bulged eye;
vi. a schematic and large mouth that overpasses the width of the nose, with thin and schematic lips, has replaced a narrow mouth.

The high modification of forms on the mask may suggest more the intentional will to change the proportions, the features and perhaps the style of the coffin rather than a simple correction of errors made by the painter during the production process ^57^. These elements could suggest a possible reuse of an older coffin, a hypothesis corroborated by the original curly wig which is characteristic of older periods and by the fact that the style of the inner lid coffin and the mummy board of the same set are typologically completely different.

### The morphometric approach. Markers, variables and types

The second part of the Faces Revealed Methodology represents the most challenging task of the project: to understand if different objects can be connected based on the way specific features have been rendered. The main objective is to identify possible common styles and trademarks of production.

This part of the work draws inspiration from the Morellian Method based on the concept that ‘incidental details of the way a particular artist portrays ears and hands, might be used to attribute unsigned paintings or sculptures to known artists’ ^58^. Following this approach, repeated patterns could correspond to habitual techniques that could be associated with a workshop or individual artist. In the decoration layer, we know that this is possible as evidenced in different studies ^59^. For example, this is clearly visible on two yellow coffins in the Museo Archeologico Nazionale di Napoli (MANN) which are linked by specific trademarks to each other and to other two yellow coffins, suggesting a common production ^60^.

As we explained, there is a huge variability in geometry, greater than in decoration, so the possibilities are numerous. Moreover, faces and features may be produced with a large amount of plaster or be sculpted on wood; the availability of resources, the type of materials, as well as the abilities of a single artist and the resources of the clientele are all elements that can have a significant impact on the final result and consequently affect this kind of study. Even if we have to consider all these elements and pay attention, it is anyway possible to link objects to each other based on specific features, as inner lid and mummy board of Amenhotep testifies (see *supra*).

But is it possible to identify markers and link coffins which are not part of the same set?

To try to answer to this question, the coffins were digitally "dissected", taking into account fixed variables, equal both in Visual appearance and Physical geometry. Seven variables were identified for Visual appearance and nine for Physical geometry ^61^. Once isolated, each variable was analysed following a morphometric approach, starting from the forms, with the measurements, to better classify shapes and to understand the proportions of each part related to the general dimension of the object.

**Figure 12.**
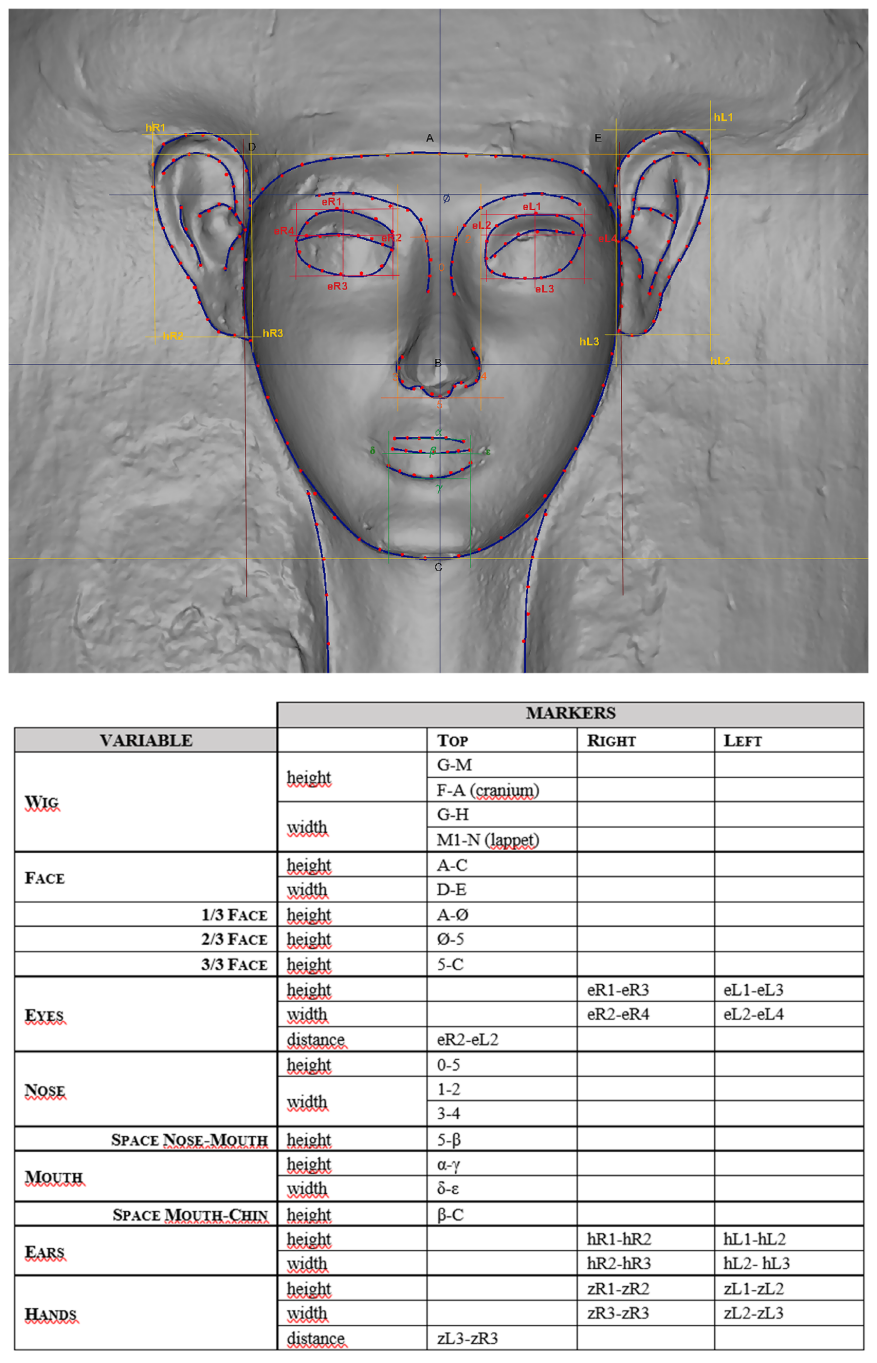
Variables and Markers for measurements. The orthophoto is courtesy and authorised by the Museo Egizio, Torino.

Measurements were taken by overlapping “layer” 2 (Physical geometry) with “layer” 4 (the “layer” that highlights geometry through points). Both were loaded and scaled into the AutoCAD software. Each object was entered onto a grid system that allows all linear measurements of the object to be controlled. The main horizontal and vertical lines of the grid intersect at the point of the nose to divide the face into approximate quarters that vary according to the face. The vertical and horizontal distances represent the variables related to the face with its four main components: eyes, ears, nose and mouth ( Figure 12) ^62^. Each variable has specific markers identified through Latin or Greek letters and/or numbers and colours, a choice that makes it easy to identify the feature and the side (upper, right or left) to which it relates.

This grid system allows to control of all the linear measurements of the object. The grid dedicated to the face, for example, allows measurement of the main distances: the distance between the midpoint of the hairline and the lowest point of the chin ( **A–C**) and the distance between the endpoints of the left and right cheeks ( **D–E**). Other horizontal lines further divide the face into 3 horizontal parts: the forehead area ( **A–Ø**); the eye and nose area ( **Ø–5**); and the mouth and chin area ( **5–C**). These measurements help to classify the forms and group faces into specific categories. Facial features are measured following the same principle: for each eye, both the distance between the outer and inner corner of the eye slit ( **eR2–eR4, eL2–eL4**), their maximum height ( **eR1–eR3, eL1–eL3**) and their distance ( **eR2– eL2**) are considered; for the nose, the maximum height from the root to the base of the nasal septum ( **0–5**) and the narrowest ( **1–2**) and largest ( **3–4**) parts; for the mouth/lips the maximum height ( **α–γ**) and the width ( **δ–ε**); for the ears the distance between the outer point of the ears, in height ( **hR1–hR2, hL1–hL2**) and width ( **hR2–hR3, hL2–hL3**). For the ears and earrings the grid system also helps to reference their position related to the face.

**Figure 13.**
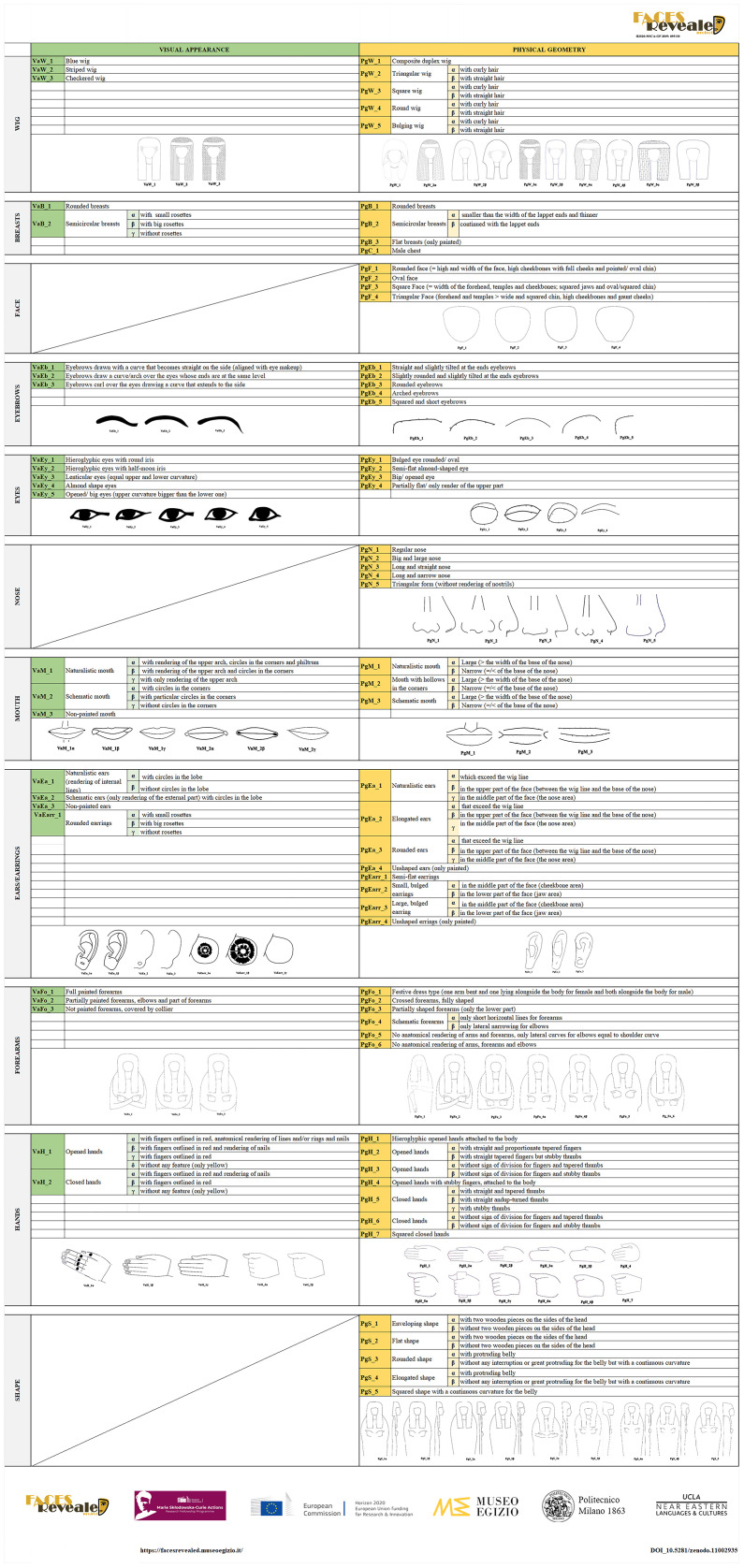
Vocabulary and types (© Faces Revealed Project).

By combining drawings, points and considering the measurements, the main variables were categorised and then, a closed vocabulary with short codes corresponding to specific types was created ^63^ ( Figure 13).

**Figure 14.**
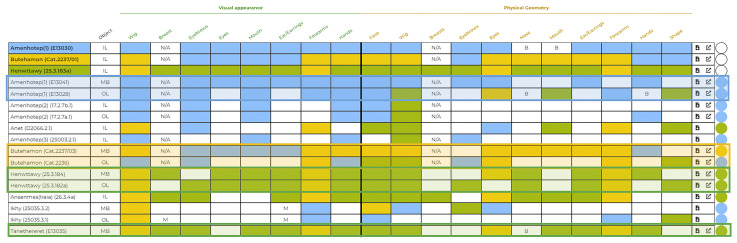
The Compare spreadsheet that highlights the common features in the coffins (© Faces Revealed Project).

This step was fundamental because allowed the construction of a Compare Spreadsheet with all the coffins and their categorised variables. The Compare Spreadsheet identifies which and how many common features the coffins share - considering both visualisations - highlighted in different coloured boxes ( Figure 14).

**Figure 15.**
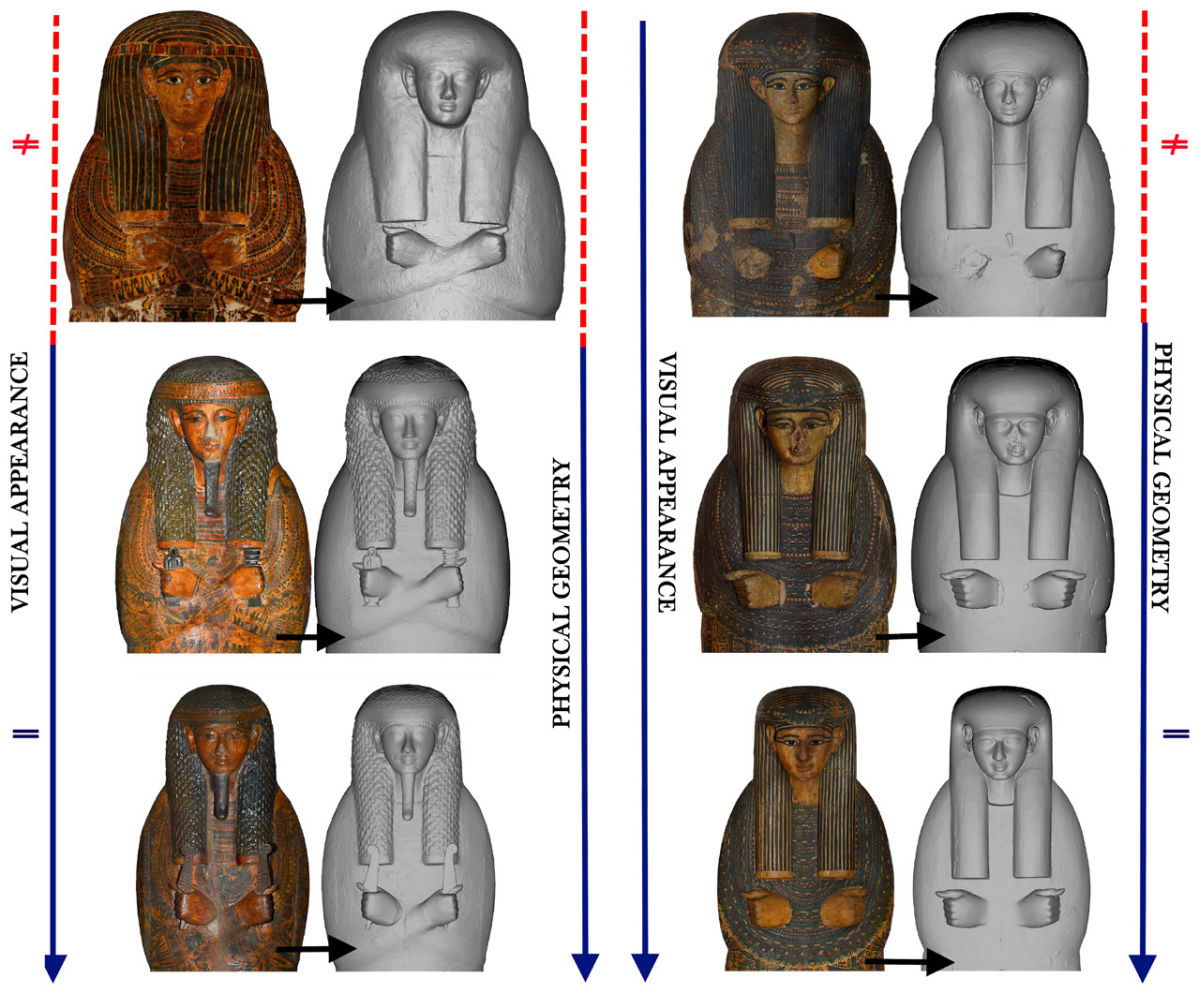
Differences and affinities in Visual appearance and Physical geometry within the coffin set of Butehamon (left) and Amenhotep (right). The orthophotos are courtesy and authorised by the Museo Egizio, Torino and the Musée du Louvre, Département des Antiquités Égyptiennes.

Using this approach it is clear, for example, that the inner lid (E13030) and the mummy board (E13041) of Amenhotep in the Louvre (in blue) have the same production, both in decoration and geometry, different from the outer lid which shares only Visual appearance and not Physical geometry features with the other two pieces, or that the outer coffin of Butehamon in Torino (Cat. 2236)(in yellow) represents a different production both in decoration and morphology with respect to its inner lid and mummy board (Cat. 2237/01-03) which are, instead, identical on both visualisations ( Figure 14 and Figure 15) ^64^.

**Figure 16.**
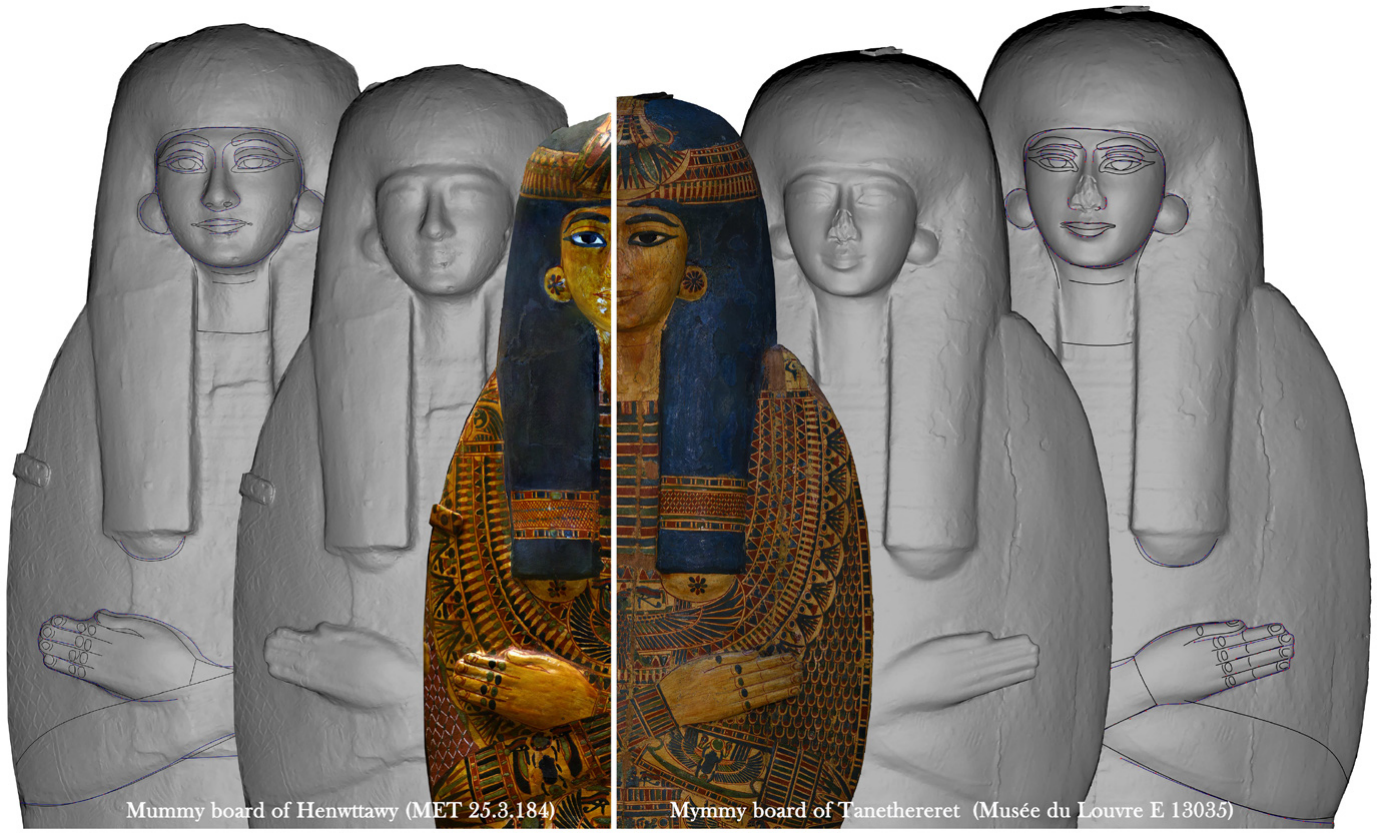
Connections between the mummy boards of Henwttawy (half right) and of Tanethereret (half left). The orthophotoa are courtesy and authorised by the Metropolitan Museum of Art, New York (MET) and the Musée du Louvre, Département des Antiquités égyptiennes.

Even if these connections and differences are visible also with the autoptic observation (see *supra* § 2.3), this categorisation allows a reliable and consistent inspection of all the variables which the comparison objective, further it enlarges the comparison itself to all the *corpus* because it enables a contemporary comparison of the variables of all the coffins, highlighting the common ones and then making it easy to identify links and differences ^65^. Among the results obtained by this method, for example, is the "discovery" of the link between the inner lid and the mummy board of Henwttawy (MET, 25.3.183a, 25.3.184), with the three pieces forming the set of Tanethereret (Louvre, E 13027, E 13034, E 13035). The way of rendering the features and the human body is the same in both Visual appearance and Physical geometry. We have, in fact, the same kind of decoration and the same "style" in the rendering of forms, evident especially in the two mummy boards ( Figure 16).

The precise connection between these objects enables one to ascribe the sets to the same production style, and speculate on a possible common "workshop"/ artist, a contemporary time of production or perhaps the existence of a “reference model” for the production of coffins in a specific place or time ^66^. These thoughts originate from the presence of a fixed type of Variables – trademarks (?) ^67^ – also on other coffin lids, all female, ascribed to the Niwinski macro-type II ^68^, and dated between the end of the 20 ^th^ and the early 21 ^st^ dynasty ^69^. Further analyses on the group are in progress, but it is clear that the Compare Spreadsheet represents an important step in the research and that an in depth analysis of features discloses abundant information about the production of coffins.

## Conclusion

The paper presents the complete developed and applied methodology of the Faces Revealed Project and introduces to the scientific community an important use of 3D models applied to the Egyptological field in an ongoing research activity ^70^.

The Faces Revealed Project is a new line of research in the Humanities where Digital Technologies and 3D models are “key tools” for research, adding new information and elements to the "traditional" research on polychrome three-dimensional objects. 3D models of yellow coffins allow us to analyse in detail the geometry and form and create orthophotos and different “layers”, enabling a much more precise analysis of the correspondence between decoration and geometry. The presence or absence of connection between the different “layers” offers an indication of the time of production of individual pieces, such as if they were produced together and, above all, whether or not they are contextual both in terms of their craft and paint, suggesting a different place of production, a possible low quality of the committee and/or “workshop” or constituting an example of reuse with different pieces being remade to match each other through decoration.

The second part of the Faces Revealed Methodology represents the most challenging task of the project: understand if the different ways of rendering shapes, ears, breasts, and mouths may be considered trademarks able to connect different coffins and place them in a specific place ("workshop") and time (chronology) of production. To identify these connections the coffins have been “dissected” and each feature forming the human body analysed and categorised to facilitate the research. As the examples of the coffin sets of Henwttawy and Tanethereret show, the most important result of this part of the methodology is the possibility to group objects and identify types not only based on Visual appearance but also on their Physical geometry giving more information about the production of yellow coffins and reconstructing a part of Ancient Egyptian history still too little known.

## Ethics and consent

Ethics and consent were not required.

## Data Availability

The Faces Revealed Project is accessible at the public page https://facesrevealed.museoegizio.it/. The raw and generated data and metadata are and will be stored in a Zenodo repository at the link ( https://zenodo.org/communities/facesrevealedprojectmsca895130?q=&l=list&p=1&s=10&sort=newest and below). Full or limited access is regulated according to individual museums' copyright restrictions. Zenodo: 3D Models of the yellow coffins in the Museo Egizio, Torino (Italy) (1.0). https://doi.org/10.5281/zenodo.10589491 ( Mainieri, 2024a). This project contains the following underlying data: 3D Models of yellow coffin lids in the Museo Egizio, Torino Zenodo: 3D Model of the Mummy board of Butehamon, Museo Egizio, Torino (Inv. n. Cat. 2237/03) (Version 2). https://doi.org/10.5281/zenodo.10722083 ( Mainieri, 2023c). 3D model of the mummy board of the Royal Scribe of the Necropolis Butehamon (Museo Egizio, Torino, Cat. 2237/03). Zenodo: 3D Model of the Inner lid of Butehamon, Museo Egizio, Torino (Inv. n. Cat. 2237/01) (Version 2). https://doi.org/10.5281/zenodo.10722825 ( Mainieri, 2023d). 3D model of the yellow coffin's inner lid of the Royal Scribe of the Necropolis Butehamon (Museo Egizio, Torino, Cat. 2237/01). Zenodo: 3D Models of the yellow coffins in the Rijksmuseum van Oudheden, Leiden. https://doi.org/10.5281/zenodo.10992877 ( Mainieri, 2024b). 3D Models of yellow coffin lids in the Rijksmuseum van Oudheden, Leiden Zenodo: 3D Models of yellow coffins in the Musée du Louvre. https://doi.org/10.5281/zenodo.11063613 ( Mainieri, 2024c). 3D Models of yellow coffin lids in the Musée du Louvre Data are available under the terms of the Creative Commons Attribution 4.0 International license (CC-BY 4.0). The following data cannot be made publicly available due to individual museums' copyright restrictions. To have access to the data please contact the author. Mainieri (2023e). 3D Models of the coffin set of Tabakmut, Metropolitan Museum of Art, New York (Version 2) [Data set]. Zenodo. https://doi.org/10.5281/zenodo.10723049 Mainieri (2024d). 3D Models of yellow coffin lids in the Museo Archeologico Nazionale di Napoli (MANN) [Data set]. Zenodo. https://doi.org/10.5281/zenodo.11002992 Mainieri (2024e). 3D models of the yellow coffins in the Museo Gregoriano Egizio, Musei Vaticani [Data set]. Zenodo. https://doi.org/10.5281/zenodo.11080925 Mainieri (2024f). 3D Models of yellow coffin lids in the Egyptian Museum in Cairo (EMC) [Data set]. Zenodo. https://doi.org/10.5281/zenodo.11003088 Mainieri (2024g). 3D Model of the yellow coffin of Padiamon in the National Museum of Egyptian Civilization (NMEC) [Data set]. Zenodo. https://doi.org/10.5281/zenodo.11003100 Mainieri (2024h). 3D Models of the Anonymous yellow coffin set in the Los Angeles County Museum of Art (LACMA) (1,0) [Data set]. Zenodo. https://doi.org/10.5281/zenodo.10590118
