# Supplementary material for: Faces Revealed Project and ancient Egyptian yellow coffins: A new methodology step-by-step
Source: Open Res Eur. 2024 Sep 3;4:193. [Version 1] doi: 10.12688/openreseurope.17421.1 (PMC11464963; doi:10.12688/openreseurope.17421.1)
Supplement: Peer review history

# Reviewer report: Reviewer response for version 1

**DOI:** 10.21956/openreseurope.18827.r44320

**Published:** 2024-11-06

**Authors:** Caroline Arbuckle MacLeod

**Affiliations:** 1 University of Saskatchewan, St Thomas More College, Saskatchewan, Canada

The Faces Revealed Project is a novel approach to the study of coffins, and represents a significant data set for Egyptologists working on funerary materials. As the author rightly notes, the Egyptological focus on coffin decoration has added considerably to our understanding of Egyptian religion, but these funerary containers have the potential to reveal much more about ancient Egyptian society, especially related to the actions of craftspeople. A particular strength of the discussion is the comparison of the visual appearance of the facial features of coffins compared to their physical geometry. The author lays out their methods for this approach carefully, demonstrating how photogrammetry can be used to create a visual representation of the coffin’s shape, free of distracting painted decoration. I think this is an excellent means of demonstrating the extent to which the painting phase of coffin creation could be used to modify the crafted face.

 The second part of the article, related to tracking hands, also has some interesting conclusions. I think the author’s detailed classification guide for different features in both shape and decoration is also very useful. I found the classification system quite complicated, and was grateful for the figures that helped to illustrate and summarize differences and affinities in visual appearance and physical geometry within specific coffin sets.

 Given that this is the first step for this project, I think it adds considerably to our knowledge. Beyond tracking artists’ hands, it would be interesting to know what additional research questions this data had inspired for the author. For instance, the author notes that details were often constructed from plaster. It would be interesting to know how many of the faces with well-aligned decoration also had considerable plaster remodeling, and/or whether the heavily repainted models lack plaster modifications. This might help indicate the types of remodeling being considered and which types of craftspeople needed to be involved.

 In total, I think this is a very useful and interesting project, and a great new means of assessing craftsmanship in Egyptian coffins. I think it opens up very exciting new approaches for research.

Is the rationale for developing the new method (or application) clearly explained?

Yes

Are the conclusions about the method and its performance adequately supported?

Yes

Is the description of the method technically sound?

Yes

Are sufficient details provided to allow replication of the method development and its use by others?

Yes

If any results are presented, are all the source data underlying the results available to ensure full access and reuse by other researchers?

Yes

Reviewer Expertise:

Egyptology, Archaeology, Wood Science, Egyptian Coffins

I confirm that I have read this submission and believe that I have an appropriate level of expertise to confirm that it is of an acceptable scientific standard.

# Reviewer report: Reviewer response for version 1

**DOI:** 10.21956/openreseurope.18827.r44319

**Published:** 2024-10-10

**Authors:** Willeke Wendrich

**Affiliations:** 1 Politecnico di Torino, Turin, Piedmont, Italy; 2 Near Eastern Languages and Cultures, University of California Los Angeles College of Letters and Science (Ringgold ID: 118546), Los Angeles, California, USA

The Faces Revealed Project provides a well-developed method to research the practices, as well as the communities of practice involved in the production of 21 ^st^ Dynasty wooden coffins, by creating detailed three-dimensional models and comparing the wooden sub-structure with the painted appearance of the coffins. In the last decade research into coffins from this period has shown that many of the 21 ^st^ dynasty coffins were a re-use of older models, which were sometimes recarved, but mostly repainted to fit the appearance that was required for the up-to-date style of this period. Hairstyles, and especially the shape of hands, breasts and the ratio of faces (location and shape of eye-brows, eyes, nose and mouth) were adapted by repainting and thus hiding the original features that can clearly be seen when the paint layer is “stripped away” from the three-dimensional model. The Faces Revealed project for the first time embarked on this research in a most thorough and systematic way. High quality three-dimensional models and a rigorous research protocol has provided a base line of important information that will be the standard for decades to come. It has replaced the purely stylistic analysis that has been the standard so far. The results of the research show that coffin sets for the same person (often consisting of mummy board, inner coffin, outer coffin) were made from various pieces that originally did not belong together, but were given a unified appearance as a “set” through overpainting. The project also enables a second research avenue that addresses the characteristics of the various ateliers or even of individual artists/artisans. The approach to this line of research is careful and well-balanced. 

 The excellent illustrations in the article display an effective use of supporting, or perhaps even making the argument.

 The project makes the full data in the form of the individual three-dimensional models available for further research.

Is the rationale for developing the new method (or application) clearly explained?

Yes

Are the conclusions about the method and its performance adequately supported?

Yes

Is the description of the method technically sound?

Yes

Are sufficient details provided to allow replication of the method development and its use by others?

Yes

If any results are presented, are all the source data underlying the results available to ensure full access and reuse by other researchers?

Yes

Reviewer Expertise:

Egyptology, archaeology, history of architecture, digital humanities

I confirm that I have read this submission and believe that I have an appropriate level of expertise to confirm that it is of an acceptable scientific standard.

# Reviewer report: Reviewer response for version 1

**DOI:** 10.21956/openreseurope.18827.r44318

**Published:** 2024-10-09

**Authors:** Mona Hess

**Affiliations:** 1 University of Bamberg, Bamberg, Germany

This article summarises the efforts to create this new corpus of a unique typology of Egyptian yellow coffins, distributed among collections around the world. Furthermore, the successful "Faces Revealed" Project has been published in an exemplary manner, including the publication of all acquired data on Zenodo, a web-based viewer-friendly publication on Sketchfab, accompanied by an informative website showing how the project has been disseminated to the public and the scientific community.

 The method used is based on 3D imaging methods with metric control and according to geomatics procedures for best image acquisition and optimised processing, taking into account the functionality for this research (calibrated targets, error margin, resolution, lighting, etc.). 3D imaging methods are now well established in cultural heritage and archaeology, as is colour or greyscale visualisation, but the proposed method of investigation, of superimposing geometries and colours for autoptic and morphometric inspection of these objects in order to gain new knowledge, is a contribution to applied science. Systematic comparisons can be facilitated and visualised with the aid of property tables. It represents a transferable, reproducible methodology that could be applied to other typologies of three-dimensional objects.

Is the rationale for developing the new method (or application) clearly explained?

Yes

Are the conclusions about the method and its performance adequately supported?

Yes

Is the description of the method technically sound?

Yes

Are sufficient details provided to allow replication of the method development and its use by others?

Yes

If any results are presented, are all the source data underlying the results available to ensure full access and reuse by other researchers?

Yes

Reviewer Expertise:

3D imaging, geomatics, cultural heritage, digital heritage

I confirm that I have read this submission and believe that I have an appropriate level of expertise to confirm that it is of an acceptable scientific standard.

# Reviewer report: Reviewer response for version 1

**DOI:** 10.21956/openreseurope.18827.r44323

**Published:** 2024-09-30

**Authors:** Andrea D'Andrea

**Affiliations:** 1 Università degli Studi di NAPOLI "L'Orientale", Naples, Italy

The paper examines the use of photogrammetry for the restitution of a collection of Egyptian painted sarcophagi. After listing and describing the category of artefacts, the author proposes a methodology for the acquisition and processing of this heterogeneous corpus of sarcophagi held in different museums. The author's goal is to examine the geometry of the sarcophagus, which is often not recognizable due to the pictorial decoration. A morphometric approach can contribute to a more correct identification of chronology and places of production. The article is a good example of how 3-D replicas can be used for scientific and not just communication purposes. The idea is very original and well carried out by the author. The contribution can be fully accepted. 

 I would recommend that all the tables and figures in the text, which often hinder easier reading, be moved to an appendix. Figure 13 is illegible. The article as presented is complete. However, I would suggest in the introduction to also describe the construction techniques and not only the history of the studies.

Is the rationale for developing the new method (or application) clearly explained?

Yes

Are the conclusions about the method and its performance adequately supported?

Yes

Is the description of the method technically sound?

Yes

Are sufficient details provided to allow replication of the method development and its use by others?

Yes

If any results are presented, are all the source data underlying the results available to ensure full access and reuse by other researchers?

Yes

Reviewer Expertise:

Archaeology, 3D data-acquisition, 3D processing

I confirm that I have read this submission and believe that I have an appropriate level of expertise to confirm that it is of an acceptable scientific standard.
